# Medium- and longer-term cardiovascular effects of e-cigarettes in adults making a stop-smoking attempt: a randomized controlled trial

**DOI:** 10.1186/s12916-022-02451-9

**Published:** 2022-08-16

**Authors:** Markos Klonizakis, Anil Gumber, Emma McIntosh, Leonie S. Brose

**Affiliations:** 1 Lifestyle Exercise and Nutrition Improvement (LENI) Research Group, Department of Nursing and Midwifery, Sheffield Hallam University, Sheffield, S10 2BP UK; 2 Centre for Sport and Exercise Science, Sheffield Hallam University, Sheffield, S10 2BP UK; 3 Institute of Psychiatry, Psychology and Neuroscience, King’s College London, London, UK; 4 SPECTRUM Research Consortium, Edinburgh, UK

**Keywords:** CVD, Smoking cessation, Vaping, Nicotine replacement therapy, Vascular function

## Abstract

**Background:**

Smoking is a major risk factor for cardiovascular disease and smoking cessation reduces excess risk. E-cigarettes are popular for smoking cessation but there is little evidence on their cardiovascular health effect. Our objective was to compare the medium- and longer-term cardiovascular effects in smokers attempting to quit smoking using e-cigarettes with or without nicotine or prescription nicotine replacement therapy (NRT).

**Methods:**

This was a single-center, pragmatic three-arm randomized (1:1:1) controlled trial, which recruited adult smokers (≥ 10 cigarettes/day), who were willing to attempt to stop smoking with support (*n* = 248).

Participants were randomized to receive behavioral support with either (a) e-cigarettes with 18 mg/ml nicotine, (b) e-cigarettes without nicotine, and (c) NRT.

Flow-mediated dilation (%FMD) and peak cutaneous vascular conductance (CVCmax) responses to acetylcholine (ACh) and sodium nitroprusside (SNP), mean arterial pressure (MAP), and other outcomes were recorded at baseline, 3, and 6 months after stopping smoking. Data were analyzed using generalized estimating equations (GEE).

**Results:**

At 3- and 6-month follow-up, %FMD showed an improvement over baseline in all three groups (e.g., *p* < 0.0001 at 6 months). Similarly, ACh, SNP, and MAP improved significantly over baseline in all groups both at 3 and 6 months (e.g., ACh: *p* = 0.004, at 6 months).

**Conclusions:**

Smokers attempting to quit experienced positive cardiovascular impact after both a 3- and 6-month period. None of the groups (i.e., nicotine-containing and nicotine-free e-cigarettes or NRT) offered superior cardiovascular benefits to the others.

**Trial registration:**

ClinicalTrials.gov NCT03061253. Registered on 17 February 2017.

**Supplementary Information:**

The online version contains supplementary material available at 10.1186/s12916-022-02451-9.

## Background

Smoking remains the second-leading mortality risk factor in the world [1], mainly due to cancer, lung disease, and cardiovascular disease (CVD). Smoking prevalence in the UK has declined substantially over the last decades to 14% in 2019 [2]. Nonetheless, smoking-caused deaths remain high (78,000 deaths in 2017 [3]), with smoking being the leading risk factor for loss of healthy life years [1].

Smoking is a major CVD risk factor. This is because it causes endothelial injury and dysfunction in both coronary and peripheral arteries and an increased risk of thrombosis. Smoking also produces a chronic inflammatory state that contributes to atherogenic disease processes and elevated levels of biomarkers of inflammation [4]. The negative effects upon cardiovascular function are not limited to smokers; passive smoking increases heart disease death-risk by almost 30% [5].

Thankfully, smoking cessation offers CVD risk-reduction benefits, which increase as time since cessation increases [6]. A combination of behavioral support and pharmacological interventions, particularly using two forms of nicotine replacement therapy (NRT) or varenicline, is the most effective approach to smoking cessation [7–9]. However, even the best methods have high relapse rates of over 75% within a year [10]. In the UK, stop-smoking services offer behavioral support and medication; however, the number of services has been reduced year on year [11].

Vaping products or electronic cigarettes (e-cigarettes) have been the most popular choice of support for smoking cessation in England, and by 2020 were being used by 27% of smokers making a cessation attempt [12], compared with for example 18% who used nicotine replacement therapy (NRT).

A recent Cochrane review concluded cessation was higher with nicotine-containing e-cigarettes than with NRT, nicotine-free e-cigarettes, or behavioral support [13]. Population-level survey data show that cessation attempts with the use of an e-cigarette had increased odds of success (OR = 1.95, 95% confidence interval (CI): 1.69–2.24) compared with those using any other support [14]. Per year, an additional 50,000–70,000 smokers in England have stopped smoking successfully, who without e-cigarettes would have continued smoking [15]. Routine monitoring data from stop-smoking services indicate that clients using e-cigarettes in their supported quit attempt are at least as likely to stop smoking as those using prescription medication [3, 12].

Nevertheless, evidence on the effect of e-cigarettes on cardiovascular health remains limited [16, 17]. Systematic reviews of e-cigarettes and cardiovascular effects suggest a negative effect [18]; however, in general, e-cigarettes are considered less harmful than traditional smoking [19].

One randomized controlled trial found that after 1-month, endothelial function and vascular stiffness improved significantly in participants who switched from smoking to using e-cigarettes compared with those who continued to smoke. The improvement was larger if participants switched to e-cigarettes completely and very similar for nicotine-containing and nicotine-free e-cigarettes [20]. Our own work on e-cigarettes’ short-term, physiological effects suggests that positive indications appear earlier in the smoking cessation time-line, and that they are similar to those offered by NRT [21]. Nevertheless, to date, no evidence has been published on the stability of improvements over time, particularly in comparison to NRT, and the range of outcome measures has been limited.

The present study therefore aimed to compare medium- (3 months) and longer-term (6 months) cardiovascular effects in smokers who attempted to quit smoking using behavioral support and (a) e-cigarettes with nicotine, (b) e-cigarettes without nicotine, and (c) prescription NRT.

## Methods

The full protocol, alongside sample size calculations, has been published previously [22].

### Design and setting

This single-center pragmatic three-arm randomized controlled trial was conducted in Sheffield, UK; recruitment took place between June 2017 and July 2019.

### Participant recruitment

Participants were recruited from the community [22]. Eligible were smokers who had smoked ≥ 10 cigarettes per day for the last year, were aged ≥ 18, and willing to make a cessation attempt using a stop-smoking service or e-cigarettes.

Exclusion criteria were (i) inability to walk; (ii) recent (within 6 months) CVD events or cardiac surgery; (iii) insulin-controlled diabetes mellitus; (iv) coexisting skin conditions, leg ulcers, vasculitis, or deep venous occlusion; (v) pregnancy; (vi) major surgery scheduled during the study; (vii) contraindications /unsuitability for NRT; (viii) current daily use of e-cigarettes; and (ix) current cessation attempt supported by a stop-smoking service.

### Procedures

Following a telephone pre-screening and information about study procedures, participants were invited to Sheffield Hallam University to provide informed consent and undertake baseline assessments. They were enrolled by a researcher not involved in group allocation, intervention delivery, or assessments. Following baseline, participants were randomized remotely into three groups by an independent statistician using a computer-generated (nQuery Advisor 6.0, Statistical Solutions, Ireland) block-randomization stratified by gender and “pack-years” (number of packs (20 cigarettes per pack) per day times number of years smoked). The study statistician allocated a unique trial number to each participant for the study duration.

Outcome assessors were blinded to group allocation and participants were reminded regularly not to share their group allocation with assessors or those providing behavioral support. The study statistician/health economist was blinded to group allocation. Those delivering the intervention were only blinded in relation to which e-cigarette group the participants belonged to as the NRT group was receiving support through the stop-smoking service. No blinding existed for participants, as NRT was delivered separately and as experienced users of nicotine, the e-cigarette groups were able to determine the presence or absence of nicotine.

During their initial behavioral support sessions, participants set a “quit date” to stop smoking completely. This defined timing of follow-up visits.

### Intervention

#### Nicotine-containing e-cigarettes group and nicotine-free e-cigarettes group

 Behavioral support was offered for a 3-month period through the research team. This was necessary as e-cigarettes were not part of the treatments offered by the local stop-smoking services at the time of the study. Both groups received complimentary e-cigarette equipment and refills (Tornado V5, Totally Wicked, Blackburn, UK), together with instructions on correct usage. Participants, on average, received 20 bottles of 10 ml during the intervention period. The nicotine-containing e-cigarette group received liquids with nicotine strength of up to 18 mg/ml, the nicotine-free e-cigarette group received nicotine-free liquids (0 mg/ml). Participants could choose ice menthol or tobacco flavor (Red Label, Totally Wicked, Blackburn, UK).

##### Prescription NRT group

Following allocation, participants were referred to Sheffield stop-smoking services for behavioral support for three months. They received money or shopping vouchers (depending on personal preference) as reimbursement for NRT prescription charges.

To ensure comparability of behavioral support provided, all groups received the same level and type of behavioral support as currently offered as standard by stop-smoking services, in the form of regular face-to-face or telephone appointments as per relevant guidelines, e.g., minimum of 6 support sessions within the 3-month period [23]. All advisors had completed the same behavioral support training. To support the successful participation of our participants, we used our “six pillars of adherence” framework (based upon “social support,” “education,” “reachability,” “small groups intervention implementation,” “reminders,” and “simplicity”). None of the participants experienced side effects due to their participation in the intervention.

### Measures

Age, gender, carbon monoxide (CO—to confirm smoking status), body mass index, blood pressure, number of cigarettes and years smoked, and physical activity measured using the SF-IPAQ [24] were recorded at baseline. Body mass index, CO, blood pressure, and physical activity assessments were repeated at 3 and 6-month follow-up.

#### Primary outcome

Macrovascular function was assessed using percentage change in flow-mediated dilation. Smoking is associated with reduced FMD. Reduced altered brachial artery FMD is an early marker for endothelial dysfunction, a CVD risk factor [6], and considered a predictor for long-term, adverse cardiovascular events [25]. FMD is a non-invasive, nitric oxide-mediated measure. Baseline scanning to assess resting vessel diameter was recorded over 3 min, following a 10-min resting period, using a Nemio XG (Toshiba, Tokyo, Japan) ultrasound machine, according to the International Brachial Artery Reactivity Task Force guidelines [26]. Fixed anatomic landmarks (side branches) were used to ensure that the same artery portion was assessed on each occasion. The technical error in our lab for FMD is 5% [26]. FMD is presented as change in post-stimulus diameter as a percentage of the baseline diameter (%FMD).

#### Secondary outcomes

Upper-body microvascular function was assessed using peak cutaneous vascular conductance (CVC) responses to acetylcholine (ACh) and sodium nitroprusside (SNP) as indicators of microvascular endothelial-dependent and endothelial-independent vasodilation, respectively, measured using Laser Doppler Fluximetry and Iontophoresis, using a standardized procedure [27].

Cutaneous red cell flux was measured by placing an iontophoresis laser Doppler probe (PF481-1; Perimed AB), connected to a laser Doppler fluxmeter (PF5001; Perimed AB), in the center of each of two drug delivery electrodes ((PF383; Perimed AB, Jarfalla, Sweden) positioned over healthy-looking skin, approximately 4 cm apart with one containing 100 μL of 1% ACh (Miochol-E, Novartis, Stein – endothelium-dependent vasodilator) and the other 100 μl of 1% SNP (Nitroprussiat, Rottapharm – endothelium-independent vasodilator). Measurements of red cell flux (recorded in arbitrary units, AU) were divided by corresponding MAP values (in mmHg) to give CVC in AU/mmHg. Here we present ACh and SNP peak CVC responses. The technical error of measurement for drug-induced peak flux responses in our laboratory is 15% [28].

Mean arterial pressure (MAP) was calculated using the following formula:

$$\frac{\left(2\times Diastolic\;pressure\right)+Systolic\;pressure}3$$

MAP was not included in the original list of outcomes as a separate outcome. However, it was decided to be analyzed and presented separately, due to its common use as surrogate marker for arterial stiffness [28]. All cardiovascular outcomes (following, micro- and macro-vascular assessments) were assessed by the same researcher, at all time-points.

Additional outcome measures include the following: (i) quality of life and health economics; preferences were calculated using EQ-5D-5L Index values and visual analog scale (VAS) scores, and the five discrete EQ-5D dimensions (mobility, self-care, usual activities, pain/discomfort, anxiety/depression) were assessed [29]; (ii) Q-Risk to assess risk of CVD; [30] and (iii) “Finger prick” test to calculate the total cholesterol (TC) over high-density lipoprotein (HDL) ratio [31].

All outcomes were assessed with participants abstaining from caffeine, main meals, supplements, and e-cigarettes and smoking for at least 3 h prior to the assessments. Premenopausal women were studied during days 1–7 of their menstrual cycle to minimize the influence of cyclical changes in female hormones and strenuous exercise was avoided for at least 12 h prior to the assessments. Outcomes were assessed at baseline and repeated at 3 and 6 months after the quit date. Resource use (e.g., GP practice and clinic visits) and personal out-of-pocket expenditure (e.g., travel for appointments, prescriptions, and products purchased to quit smoking) were collected via participants’ diaries maintained from baseline to the end of intervention (3 months) period using standard procedures.

### Sample

**Fig. 1:**
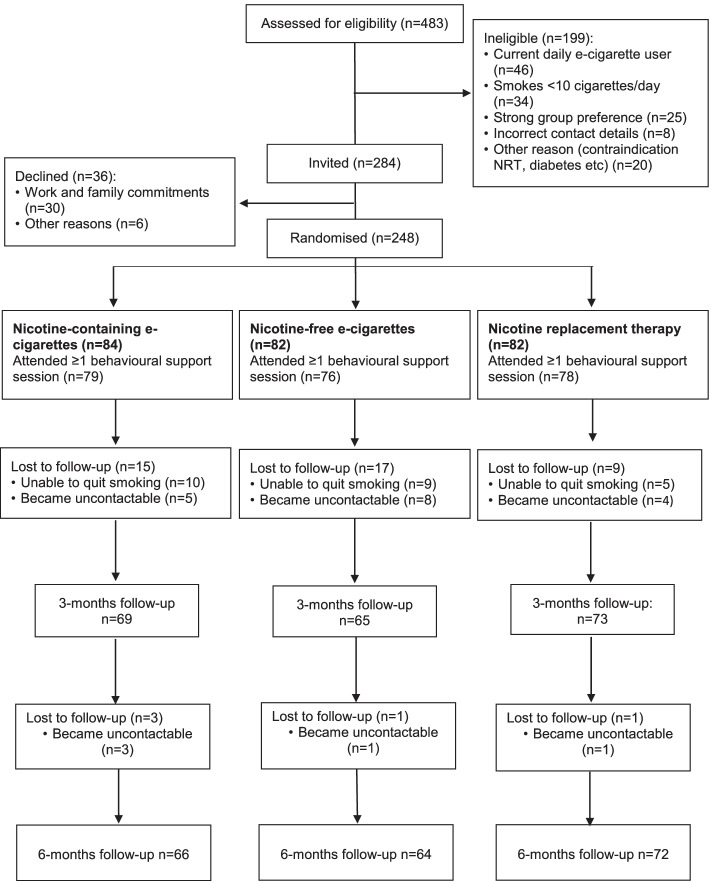
Flowchart of the study population

In total, 248 participants were randomized (Fig. 1). Data of four were removed following their withdrawal from the study (1 in the nicotine-containing, 1 in the nicotine-free, 2 in the NRT group). The 3-month follow-up was completed by 207 participants, the 6-month follow-up by 202 participants. Participants were included whether they stopped smoking or not.

### Statistical analysis

Data were analyzed in SPSS 24 (IBM U.K. Limited, Hampshire, UK) on an intention-to-treat basis, using the “last observation carried forward” method for those participants that had no data for the 3- and 6-month follow-ups, respectively.

We developed a multiple linear regression model for our repeated continuous outcome measures (%FMD, ACh, SNP, MAP), using a generalized estimating equations (GEE) approach. We made this change from the ANOVA specified in the protocol as GEE is more robust to misspecification of the dependency structure, which results from the repeated assessments of individual over time [32]. GEE used age (in completed years), gender and baseline measurements (or 3-month values for change over 6-month analysis) to control for and predict net-changes in these outcome measures over the 3- and 6-month periods, across our three randomized groups. To explore the interaction between our repeated assessments and our treatment groups, we introduced treatment groups dummy variables, which were assessed over each time-point (i.e., 3 and 6 months), using an established methodology as described in Twisk et al. [33]. Our analysis was also varied against our original protocol, in that we decided against including pack years in the model, due to the excellent balance between groups; age was included instead. A separate analysis using mixed effect models repeat measurement (MMRM) was completed after using multiple imputation for missing values for four outcome variables at 3- and 6- month follow-ups; findings did not differ to those obtained through the GEE modeling.

As additional post hoc analyses, separate GEE models were run for the “cessation” sub-group, which included those that stated that they had stopped smoking and had their status biochemically validated by exhaled air measurement of < 10 ppm CO in line with the Russell Standard [34].

Unadjusted mean differences (d) were calculated as the mean change from baseline to a given time point, for the %FMD, ACh, SNP, and MAP.

For health-related quality of life (HRQoL), mean values for the three participant groups were compared, and the net changes from baseline to 3 and 6 months were estimated. Also, the proportion of patients with sub-optimal levels (i.e., levels 2–5) on each of the five dimensions was compared at baseline, 3, and 6 months. Quality-adjusted life-years (QALYs) were calculated by multiplying the duration of time spent in various health states by the HRQoL weight (i.e., utility score) associated with that health state. A similar approach (i.e., paired *t*-tests used to compare differences in means at two points in time (3 and 6 months over baseline values) across three quit-smoking groups) was chosen for the other secondary outcomes, including Q-Risk and the total cholesterol (TC) over high-density lipoprotein (HDL) ratio.

Health economics were assessed using participants’ resource use diaries alongside costs for the delivery of 12 weeks of behavioral change intervention (researcher’s time, room hire charges, and travel expenses), and costs for the intervention materials (electronic cigarette, adapter, atomizer & liquids). NHS smoking cessation delivery costs were estimated to include clinic nurse time and the number of sessions delivered to NRT participants. Participants’ use of NHS services was priced using 2020 PSSRU costings or Department of Health reference costs [35].

### Role of the funding source

The trial was funded by Heart Research UK under a Translational Research Grant (RG2658). The funder had no role in the study’s design, conduct, and reporting.

## Results

### Participants

Baseline characteristics are presented in Table 1.

**Table 1:** Baseline characteristics of participants (*n* = 248)

Baseline characteristics	Nicotine replacement therapy (NRT), *n* = 82	Nicotine-containing e-cigarettes, *n* = 84	Nicotine-free e-cigarettes, *n* = 82
Sex, %male	44%	55%	50%
Age, mean years (SD)	44 (13)	44 (14)	44 (13)
BMI, Mean (SD)	26.5 (5.1)	27.5 (5.9)	27.4 (5.4)
Systolic blood pressure, mmHg (SD)	132 (17)	132 (18)	131 (18)
Diastolic blood pressure, mmHg (SD)	81 (12)	83 (12)	82 (14)
MAP, mean mm Hg (SD)	98 (11)	98 (11)	97 (12)
Smoking tears, mean (SD)	25(13)	24 (13)	25 (13)
Cigarettes per day, mean (SD)	18 (7)	18 (7)	16 (7)
Smoking pack years, mean (SD)	24 (19)	23 (17)	22 (17)
Exhaled air carbon monoxide (CO), mean parts per million (SD)	16.9 (6.9)	15.2 (7.9)	14.3 (8.1)
Physical activity, mean weekly MET minutes (SD)	2756 (2627)	2772 (2669)	3082 (3191)
%FMD, mean (SD)	6.7 (4.0)	6.2 (4.4)	5.6 (3.6)
Artery diameter (pre-inflation), mean (SD)	4.22 (0.66)	4.13 (0.65)	4.10 (0.56)
Artery diameter (post-inflation), mean (SD)	4.47 (0.64)	4.34 (0.61)	4.36 (0.55)
Peak CVC for ACh, mean PU/mmHg (SD)	1.34 (1.00)	1.48 (1.02)	1.49 (0.98)
Peak CVC for SNP, mean PU/mmHg (SD)	1.35 (0.98)	1.45 (1.02)	1.15 (0.72)
Total cholesterol, mmol/l (SD)	4.4 (1.0)	4.5 (1.1)	4.7 (1.2)
Total cholesterol/HDL ratio (SD)	4.4 (1.9)	4.1 (1.7)	4.1 (1.9)
FTND, points (SD)	6 (2)	6 (2)	6 (2)
Q-Risk, % (SD)	7.5 (8.7)	8.6 (10)	7.8 (9.1)
Q-Risk – Heart Health, years (SD)	53 (16)	54 (16)	54 (15)

*ACh*, acetylcholine; *BMI*, body mass index; *CVC*, peak cutaneous vascular conductance; *%FMD*, percentage change in flow-mediated dilation; *FTND*, Fagerstrom Test for Nicotine Dependence; *HDL*, high-density lipoprotein; *MAP*, mean arterial pressure; *MET*, metabolic equivalent; *SNP*, sodium nitroprusside

### Primary outcome: macrovascular assessment

**Fig. 2:**
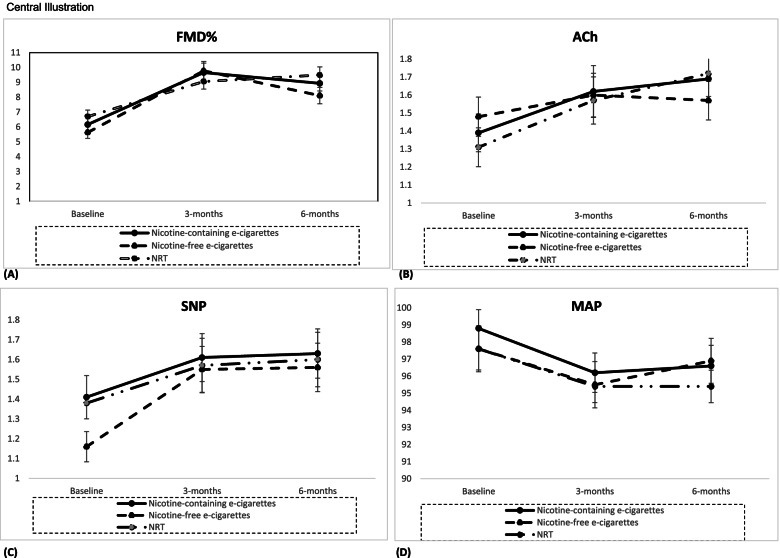
Central illustration: Changes from baseline to follow-up by treatment arm in **(A)**: Flow mediated dilation (%FMD); **(B)**: Peak cutaneous vascular conductance responses to acetylcholine (ACh); **(C)**:Peak cutaneous vascular conductance responses to sodium nitroprusside (SNP); **(D)**: Mean arterial pressure (MAP)

Compared to baseline, %FMD was improved at both 3- and 6-month follow-ups in all three groups. There was an improvement in %FMD at 3 months (β=3.33; 95% CI: 2.61 to 4.05, *p* < 0.0001) and at 6 months (β=2.69; 95% CI: 2.02 to 3.35, *p* < 0.0001). There was no statistically significant difference in %FMD between the intervention groups (Table 2, Fig. 2 Central Illustration Graph A). Age did not appear to affect our findings, although gender did, as females had a greater gain in macrovascular function improvement than males (β=-1.32, 95% CI:−2.24 to−0.4; *p*=0.005). There was also a significant combined effect of group and follow-up visits (β=3.17, 95% CI: 1.37 to 3.59; *p* = 0.001).

**Table 2:** Results of model estimating change in % flow-mediated dilation, cutaneous vascular conductance in response to acetylcholine and to sodium nitroprusside, and mean arterial pressure at 3 and 6 months follow-up: all participants

Variables	Group/follow-up	B-coeff	Exp (B)	95% CI		*p* value
				**Lower**	**Upper**	
**%FMD**						
Group	NRT over nicotine-containing e-cigarettes	0.43	1.56	− 0.64	1.50	0.43
	Nicotine-free e-cigarettes over nicotine-containing e-cigarettes	− 0.59	0.56	− 1.76	0.59	0.33
Follow-up	3 m over baseline	3.33	27.90	2.61	4.05	0.001
	6 m over baseline	2.69	14.67	2.02	3.35	0.001
Gender	Male over female	− 1.32	0.27	− 2.24	− 0.40	0.005
Age		− 0.02	0.98	− 0.05	0.01	0.23
Intercept		9.89		8.29	11.5	0.001
**Peak CVC for Ach**						
Group	NRT over nicotine-containing e-cigarettes	− 0.03	0.97	− 0.26	0.20	0.80
	Nicotine-free e-cigarettes over nicotine-containing e-cigarettes	− 0.01	0.99	− 0.25	0.23	0.93
Follow-up	3 m over baseline	0.20	1.27	0.01	0.40	0.04
	6 m over baseline	0.27	1.31	0.09	0.45	0.004
Gender	Male over female	− 0.02	0.99	− 0.21	0.18	0.88
Age		− 0.01	0.99	0.994	0.01	0.17
Intercept		1.79		1.36	2.23	0.001
**Peak CVC for SNP**						
Group	NRT over nicotine-containing e-cigarettes	− 0.03	0.98	− 0.26	0.29	0.83
	Nicotine-free e-cigarettes over nicotine-containing e-cigarettes	− 0.14	0.87	− 0.37	0.09	0.23
Follow-up	3 m over baseline	0.26	1.52	0.10	0.42	0.001
	6 m over baseline	0.28	1.57	0.11	0.45	0.002
Gender	Male over female	− 0.07	0.93	− 0.26	0.12	0.48
Age		− 0.01	1.00	0.995	0.01	0.16
Intercept		1.63		1.23	2.03	0.001
**MAP**						
Group	NRT over nicotine-containing e-cigarettes	− 1.51	0.22	− 4.24	1.23	0.28
	Nicotine-free e-cigarettes over Nicotine-containing e-cigarettes	0.28	1.33	− 2.88	3.44	0.861
Follow-up	3 m over baseline	− 1.74	0.18	− 2.57	− 0.91	0.001
	6 m over baseline	− 1.41	0.25	− 2.31	− 0.51	0.002
Gender	Male over female	4.33	75.55	1.86	6.79	0.001
Age		0.23	1.26	1.15	1.39	0.001
Intercept		9.54		7.92	11.17	0.001

*ACh*, acetylcholine; *CVC*, peak cutaneous vascular conductance; *%FMD*, percentage change in flow-mediated dilation; *MAP*, mean arterial pressure; *SNP*, sodium nitroprusside

Unadjusted mean differences from baseline (*d*) were high for all groups both at 3 (d = 4.14, 95% CI: 2.57 to 5.51, *p* < 0.001 for the nicotine-free e-cigarette group, d = 3.50, 95% CI: 2.28 to 4.72 for the nicotine-containing e-cigarette group, *d* = 2.36, 95% CI: 1.17 to 3.56, *p* < 0.001, for the NRT group) and 6 months (*d* = 2.48, 95% CI: 1.35 to 3.62 for the nicotine-free e-cigarette group, *d* = 2.78, 95% CI: 1.66 to 3.90, *p* < 0.001 for the nicotine-containing e-cigarette group, *d* = 2.79, 95% CI: 1.52 to 4.06 for the NRT group).

### Secondary outcomes: microvascular assessment

#### Acetylcholine (ACh)

Similar to %FMD, CVC values for ACh improved significantly over baseline in all three groups both at 3 and 6 months. There was an improvement at 3 (β=0.2, 95% CI: 0.01 to 0.4, *p* = 0.04) and 6 months (β=0.27, 95% CI: 0.09 to 0.45, *p* = 0.004). There were no statistically significant differences between groups at follow-up (Table 2). Neither age nor gender affected the outcome in endothelial-dependent microvascular vasodilation (Table 2; Fig. 2 Central Illustration Graph B). Nevertheless, the combination of group and follow-up visits did (β=0.32, 95% CI: 0.23 to 0.4; *p* = 0.03).

Unadjusted d values were high, particularly at 6 months (*d* = 0.19, 95% CI: − 0.08 to 0.41, *p* = 0.29 for the nicotine-free e-cigarette group, *d* = 0.30, 95% CI: − 0.01 to 0.61, *p* = 0.04 for the Nicotine-containing e-cigarette group, *d* = 0.41, 95% CI: 0.08 to 0.74, *p* = 0.02 for the NRT group).

#### Sodium nitroprusside (SNP)

As with the previous outcomes, SNP improved over baseline, at both follow-up visits. There was an improvement at both 3 (β=0.26, 95% CI: 0.09 to 0.42, *p* = 0.001) and 6 months (β=0.28, 95% CI: 0.11 to 0.45, *p* = 0.002), with the combination of group and visit bearing an effect to the outcome (β=0.49, 95% CI: 0.15 to 0.67, *p* = 0.002). There were no significant differences between groups (Table 2; Fig. 2 Central Illustration Graph C) and neither age nor gender significantly affected this outcome.

The positive improvement trend observed in other outcomes was also observed for SNP in unadjusted d as well, particularly at the 6-month follow-up: (*d* = 0.40, 95% CI: 0.13 to 0.65, *p* = 0.004 for the nicotine-free e-cigarette group, *d* = 0.22, 95% CI: − 0.08 to 0.52, *p* = 0.14 for the nicotine-containing e-cigarette group, *d* = 0.21, 95% CI: − 0.55 to 0.12, *p* = 0.21 for the NRT group).

#### Mean arterial pressure (MAP)

MAP readings were reduced in all three intervention groups at both follow-ups (*β* =  − 1.74, 95% CI: − 2.57 to − 0.91; *p* = 0.001 and *β* =  − 1.41, 95% CI: − 2.31 to − 0.51; *p* = 0.002). There were no statistically significant differences between groups (Table 2, Fig. 2 Central Illustration Graph D) and no combined effect of group and follow-up visit (β=0.26, 95% CI:−1.22 to 1.74; *p* = 0.73). The reduction was affected by gender (*β* = 4.33, 95% CI: 1.86 to 6.79; *p* = 0.001) and age (*β* = 0.23, 95% CI: 0.14 to 0.33; *p* = 0.001), with females and lower age groups benefiting the most.

As with the other measures, unadjusted *d* values suggest an improvement particularly at 6 months (*d* =  − 0.26, 95% CI: − 1.24 to 1.79, *p* = 0.53 for the nicotine-free e-cigarette group, *d* =  − 2.23, 95% CI: − 3.74 to − 0.71, *p* = 0.004 for the nicotine-containing e-cigarette group, *d* =  − 2.18, 95% CI: − 3.86 to − 0.49, *p* = 0.01 for the NRT group).

### Cessation subgroup

In the nicotine-containing e-cigarette group, 39 successfully stopped smoking at 3 and 36 at 6 months. In the nicotine-free e-cigarette group, it was 34 and 30 at 3 and 6 months, respectively; and in the NRT group 33 and 25 at 3 and 6 months respectively. The post hoc subgroup analysis showed strong effects for %FMD at both follow-up sessions (*β* = 2.40, 95% CI: 1.66 to 3.14; *p* < 0.0001 for 3 months and *β* = 2.99, 95% CI: 2.22 to 3.77; *p* < 0.0001 for 6 months). No differences were observed between groups at both time-points (Table 3).

**Table 3:** Results of model estimating change in % FMD at 3- and 6-month follow-ups of cessation subgroup (upper section) and changes in means (difference over baseline) in %FMD for the cessation subgroup for 3- and 6-month follow-ups (lower section)

Variables	Group/follow-up					B-coeff	Exp (B)		95% CI		*p* value	
									**Lower**	**Upper**		
**%FMD**												
Group	NRT over EC nicotine-containing					0.46	1.61		− 0.59	1.54	0.38	
	EC nicotine-free over EC nicotine-containing					− 0.57	0.57		− 1.74	0.60	0.34	
Follow-up	3 m over baseline					2.40	20.04		1.66	3.14	< 0.0001	
	6 m over baseline					2.99	11.04		2.22	3.77	< 0.0001	
Gender	Male over female					− 1.35	0.26		− 2.27	− 0.43	0.004	
Age						− 0.02	0.98		− 0.05	0.01	0.27	
Intercept						7.67			5.92	9.42	< 0.0001	
**%FMD changes over baseline**	**NRT**				**EC—nicotine-containing**				**EC – nicotine-free**			
	**Mean difference**	**95% CI**		***p* value**	**Mean difference**	**95% CI**		***p* value**	**Mean difference**	**95% CI**		***p* value**
		**Lower**	**Upper**			**Lower**	**Upper**			**Lower**	**Upper**	
**3 months over baseline**												
Cessation subgroup	2.61	0.97	4.25	0.003	3.94	1.73	6.15	0.001	4.78	2.27	7.29	0.001
**6 months over baseline**												
Cessation subgroup	4.40	2.18	6.66	0.001	2.96	1.17	4.74	0.002	2.97	0.90	5.04	0.006

*%FMD*, percentage change in flow-mediated dilation

Unadjusted mean differences from baseline (*d*) were high for all groups both at 3 (e.g., *d* = 4.78, 95% CI: 2.27 to 7.29, *p* < 0.0001 for the nicotine-free e-cigarette group) and 6 months (e.g., *d* = 4.40, 95% CI: 2.14 to 6.66, *p* = 0.001 for the NRT group) (Table 3).

### Secondary outcomes: other measures

For Quality of life, post-quit improvements were observed in QALYs, both in % and actual values [e.g., *F* = 4.17, *p* = 0.02, df = 2,243 at 3 and *F* = 5.03, *p* = 0.01, df = 2,243 at 6 months respectively for QALY (%)]. Improvements were also detected for pain (%) (*F* = 3.43, *p* = 0.03, df = 2,243 at 3 months) and depression and anxiety (%) (*F* = 4.54, *p* = 0.01, df = 2,243 at 3 and *F* = 6.25, *p* = 0.01, df = 2,243 at 6 months respectively). No changes were detected in mobility (%), movement (%), self-care (%) or the participants’ self-rated health (VAS) (Table 4).

**Table 4:** Quality of life by domain at baseline, 3 and 6 months follow-ups of all participants

Baseline, 3 and 6 months, mean	Nicotine replacement therapy	Nicotine-containing e-cigarettes	Nicotine-free e-cigarettes	*F* value, *p* value, and degrees of freedom
**Mobility (%)**				
Baseline	9.88	15.48	13.92	*F* = 0.60, *p* = 0.55, df = 2,243
3 months	7.41	9.52	10.13	*F* = 0.20, *p* = 0.82, df = 2,243
6 months	6.17	7.14	8.86	*F* = 0.21, *p* = 0.81, df = 2,243
**Self-care (%)**				
Baseline	2.47	5.95	3.80	*F* = 0.65, *p* = 0.52, df = 2,243
3 months	2.47	2.38	1.27	*F* = 0.12, *p* = 0.84, df = 2,243
6 months	1.23	3.57	2.53	*F* = 0.20, *p* = 0.82, df = 2,243
**Usual activity (%)**				
Baseline	8.64	16.67	18.99	*F* = 1.89, *p* = 0.15, df = 2,243
3 months	7.41	14.29	12.66	*F* = 1.04, *p* = 0.36, df = 2,243
6 months	6.17	15.48	10.13	*F* = 1.90, *p* = 0.15, df = 2,243
**Pain (%)**				
Baseline	35.80	39.29	46.84	*F* = 1.05, *p* = 0.35, df = 2,243
3 months	23.46	25.00	29.11	*F* = 0.35, *p* = 0.70, df = 2,243
6 months	17.28	21.43	34.18	*F* = 3.43, *p* = 0.03, df = 2,243
**Depression/anxiety (%)**				
Baseline	38.27	45.24	51.90	*F* = 1.50, *p* = 0.22, df = 2,243
3 months	23.46	42.86	43.04	*F* = 4.54, *p* = 0.01, df = 2,243
6 months	24.69	50.00	44.30	*F* = 6.25, *p* = 0.01, df = 2,243
**QALY (%)**				
Baseline	41.98	34.52	32.91	*F* = 0.81, *p* = 0.45, df = 2,243
3 months	62.96	44.05	43.04	*F* = 4.17, *p* = 0.02, df = 2,243
6 months	64.20	42.86	43.04	*F* = 5.03, *p* = 0.01, df = 2,243
**VAS (Mean, SD)**				
Baseline, mean (SD)	71.59 (15.98)	67.26 (15.24)	70.96 (17.14)	*F* = 1.74, *p* = 0.18, df = 2,243
3 months, mean (SD)	72.38 (14.33)	70.91 (14.42)	69.81 (19.24)	*F* = 0.51, *p* = 0.60, df = 2,243
6 months, mean (SD)	75.38 (13.80)	70.29 (16.12)	72.78 (16.22)	*F* = 2.24, *p* = 0.11, df = 2,243

Reductions were observed for Q-Risk at 3 and 6 months for all three groups with the exception of the Q-risk % at 6 months for NRT. No statistically significant changes were observed for TC/HDL Ratio (Table 5).

**Table 5:** Change of secondary outcome variables over baseline

Variables—change over baseline	Nicotine replacement therapy (NRT)				Nicotine-containing e-cigarettes				Nicotine-free e-cigarettes			
	**Mean difference**	**95% CI**		***p* value**	**Mean difference**	**95% CI**		***p* value**	**Mean difference**	**95% CI**		***p* value**
		**Lower**	**Upper**			**Lower**	**Upper**			**Lower**	**Upper**	
**CO (reduction in parts per million)**												
**3 months over baseline**	10	8	11	< .001	9	7	11	< .001	7	5	9	< .001
**6 months over baseline**	8	6	10	< .001	9	7	11	< .001	6	4	8	< .001
**TC/HDL ratio (reduction)**												
**3 months over baseline**	0.89	− 0.16	1.91	0.09	0.18	− 0.02	0.39	0.08	0.11	− 0.14	0.36	0.39
**6 months over baseline**	0.78	− 0.27	1.80	0.15	0.23	0.06	0.40	0.009	0.01	− 0.23	0.24	0.97
**QRISK (reduction in %)**												
**3 months over baseline**	1.8	0.9	2.7	< .001	1.6	0.6	2.6	0.002	2.1	1.2	3.0	< .001
**6 months over baseline**	0.9	− 0.4	2.3	0.164	1.8	0.8	2.7	< .001	1.7	0.7	2.7	0.001

Our health economics’ assessment suggests a lower per-participant cost for e-cigarettes (nicotine-containing = £192.55, nicotine-free = £177.63) in comparison to NRT (£246.92), due to a higher resource-use cost observed in the NRT group (*Please see *Online Supplement).

## Discussion

Participants improved their cardiovascular health prospects, irrespective of whether they used NRT or e-cigarettes with or without nicotine. These improvements appear both overall and in those that quit successfully.

Most of our findings (driven by the improvements in those that quit successfully, as our sub-group analysis shows) suggest endothelial function improvements, which appear quite early in the smoking cessation timeline [21], continue to exist in both smaller and larger conduits, at least up to 6 months after smokers quit. This potentially happens via a reduction in the production of oxidative stress and ultimately an increase of NO bioavailability, an imbalance between which is known to be created via smoking [36]. ACh is an endothelial-dependent vasodilator primarily associated with an increase in NO bioavailability, particularly at higher ACh doses [27]. Additionally, endothelium-derived NO is considered as a principal FMD mediator [26], although this view is debated [27].

Considering that SNP is a direct NO donor [27], our findings suggest that smoking cessation offers further NO-bioavailability benefits through an improvement in smooth muscle cell vascular function, as the observed improvement in SNP-induced vasodilation suggests. This is a change from our short-term observations, in which we found no improvements from the baseline, in any of our groups [25]. Therefore, it is possible that although e-cigarettes are known to have a negative acute effect on NO-bioavailability and endothelial function of healthy adults [37] or when comparing e-cigarette users with healthy adults [38], they appear to support the reversal of the smoking-induced endothelial dysfunction, in a similar manner to NRT. This happens, however, at a later stage of the smoking cessation process, suggesting that the smooth muscle cell damage, which is caused by cigarettes (possibly via inducing a pro-inflammatory/matrix remodeling phenotype in smooth muscle cells [39]), is more persistent. The slow reversal is a positive sign, which could also reduce the risk of atherosclerosis and stroke [39].

Although the assessment of vascular stiffness is a complex process, and a simple, surrogate measure cannot fully determine whether a change occurs or not, recent work suggests the predictive role of MAP for arterial stiffness [39], with increases in the former suggesting also an increase to the latter [40]. Thus, the observed MAP reduction in all three trial groups may well suggest an improvement in vascular stiffness as well. Direct testing, which was not possible in our study, due to the high participant burden of the assessments that we used, can confirm our findings.

Our findings agree with recent work by George et al. [20], who reported that endothelial function and arterial stiffness in smokers improved within 1 month of switching to e-cigarettes. Our work goes some steps further, showing that endothelial function is markedly improved in small veins and arteries, if smokers continue to abstain, for longer periods, from cigarettes, using NRT as well. This is an important finding, which can be potentially encouraging for those who wish to stop smoking, especially as impaired vascular function—even at pre-clinical groups—is associated with a higher CVD risk [41]. Stronger improvement in women than men found both by George et al. [20] and in the present study, indicate that physiological reasons should be further explored.

In line with previous evidence [42, 43] a lower age was associated with marginally larger beneficial changes, highlighting that while it is never too late to stop smoking, the earlier this happens, the easier it is to reverse the negative effects of cigarettes.

Finally, our Health Economics analysis suggests a lower cost for e-cigarettes to that required for NRT (£192.55 and £177.63 vs £246.92). QoL findings confirm previously reported benefits of smoking cessation [20] including among people using e-cigarettes [20].

### Limitations

There are many different devices and liquid manufacturers and a wide range of liquids available. We chose a single device and manufacturer to ensure consistency and standardization. We did not include a group of continuing cigarette smokers as it would not have been ethical to do due to the dangers of continued smoking. However, all participants were smokers initially and therefore de-facto acted as controls for themselves, while no improvements are to be expected over time in the vasculature of smokers [20]. Finally, the trial was not designed to perform a “quitters” vs “continuous smokers” sub-group comparison, which would have added to our knowledge on the topic. This should be the focus of a future study.

### Strengths

This is the first study to compare the medium- and longer-term cardiovascular effects of smoking cessation attempts supported using e-cigarettes and NRT and is being complimented by an exploration of the participants' experiences in the intervention, published separately [44]. Our intervention was conducted in a way which would be followed if e-cigarettes were widely adopted as a smoking cessation aid, in supported smoking cessation attempts. It enabled comparison with a licensed and widely accepted route for smoking cessation. The success rate of our intervention (which can be attributed to the flexibility of the behavioral support offered, and the intense way that the intervention was delivered), although it cannot be traced for a period longer than 6 months, suggests the need of healthcare investment in these services. Finally, the inclusion of micro-circulation outcomes is another strength as pathological changes in the microcirculation mirror changes in arteries, making it an appropriate area to study to achieve early detection.

### Future research

The next step would be to explore outcomes beyond 6 months. It would also be interesting to explore the cardiovascular effects of different devices and liquids, although long-term stability would likely be difficult to achieve.

## Conclusions

Smokers attempting to quit experienced a positive cardiovascular impact after both a 3- and 6-month period. None of the groups (i.e., nicotine-containing and nicotine-free e-cigarettes or NRT) offered superior cardiovascular benefits to the others.

## Supplementary Information

**Additional file 1.**

## Data Availability

The datasets generated and/or analyzed during the current study are not publicly available due to ethical approval restrictions but are available from the corresponding author on reasonable request.

## Abbreviations

ACh: Acetylcholine
BMI: Body mass index
CVC: Cutaneous vascular conductance
CVD: Cardiovascular disease
E-cigarettes: Electronic cigarettes
FMD: Flow-mediated dilation
MAP: Mean arterial pressure
NRT: Nicotine replacement therapy
SNP: Sodium nitroprusside
