# Supplementary material for: Medium- and longer-term cardiovascular effects of e-cigarettes in adults making a stop-smoking attempt: a randomized controlled trial
Source: BMC Med. 2022 Aug 16;20:276. doi: 10.1186/s12916-022-02451-9 (PMC9380327; doi:10.1186/s12916-022-02451-9)
Supplement: Supplementary file 1 — Additional file 1. [file 12916_2022_2451_MOESM1_ESM.docx]

| **Cost of Stop Smoking Intervention Delivered by Study / Stop Smoking Services and Resource Use Costs over 12 Weeks** | | | | |
| --- | --- | --- | --- | --- |
| **Costing items for intervention** | **Cost Unit/Type** |  | |  |
|  |  | **Nicotine-containing e-cigarettes** | **Nicotine-free-cigarettes** | **Nicotine Replacement Therapy** |
| **I. Stop Smoking Intervention (12 weeks):** |  |  |  |  |
| 1. Pre- Quit: Total Number of sessions for each group |  | 157 | 150 | 0 |
| 1a. Of which telephonic sessions |  | 47 | 62 |  |
| 2b. Of which Face to Face sessions |  | 110 | 88 |  |
| 2. Pre- Quit: Patient contact time of the Researcher (30 minutes) | £20 per hour | £1,570 | £1,500 | 0 |
| 3. Pre- Quit: Room hire cost: Face to Face | £9 per hour | £495 | £396 | £729 |
| 4. Pre- Quit: Travel cost voucher per participant | £ | 0 | 0 | 0 |
| 5. Pre- Quit: Refreshment during session per participant | £ | 0 | 0 | 0 |
| 6. Post- Quit: Total Number of sessions for each group |  | 219 | 215 | 0 |
| 6a. Of which telephonic sessions |  | 79 | 84 |  |
| 6b. Of which Face to Face sessions | £ | 140 | 131 |  |
| 7. Post- Quit: Patient contact time of the Researcher (30 minutes) | £20 per hour | £2,190 | £2,150 | 0 |
| 8. Post- Quit: Room hire cost: Face to Face (if Any) | £9 per hour | £630 | £589.50 | 0 |
| 9. Post- Quit: Refreshment during session per participant | £ | 0 | 0 | 0 |
| 10. Stop Smoking Services Clinic Attendance | Number |  |  | 342 |
| 10.1 Pre-quit Session cost (2 sessions, 45 minutes each) | £40 per hour |  |  | £4,860.00 |
| 10.2 Remaining follow-up sessions (30 minutes each) | £20 per session |  |  | £3,560.00 |
| **Total Cost of Behavioural Support Intervention** | **£** | **£4,885.00** | **£4,635.50** | **£9,149.00** |
| **II. Intervention Supplies (For 12 Weeks of Intervention)** |  | **84** | **82** | **82** |
| 1. E-cigarettes received | £24 per EC | £2,064.00 | £1,968.00 |  |
| 2. Adapters/chargers | £1.50 x 2 boxes | £258.00 | £246.00 |  |
| 3. Atomizers | £5 x 1 | £430.00 | £410.00 |  |
| 4. Ice Menthol (1.8%)/ Tobacco (1.8%) bottles | £2.80 x 23 | £5,538.40 | 0 |  |
| 5. Ice Menthol (0%) / Tobacco (0%) bottle | £2.80 x 23 | 0 | £5,280.80 |  |
| 6. Any Replacement of above faulty kit (EC, Adapter, Atomizer & liquids) | £96.4 per kit | £289.20 | £385.60 |  |
| **Total Cost of Supplies** |  | **£8,579.60** | **£8,290.40** | **£0.00** |
| 7. NHS Net Prescription Kit Cost (borne for supplies to NRT person (£25-£9=£14) x 3 times | £9/£14 | £9.00 | £0.00 | £3,444.00 |
| 8. Out-of-pocket purchases |  | £0.00 | £15.50 | £135.00 |
| **III. Total Cost of Stop Smoking Intervention Delivery** |  | **£13,473.60** | **£12,941.40** | **£12,728.00** |
| IV.1. Health professionals (visits at least partially related to stop smoking attempt |  |  |  |  |
| GP-Doctor | £43.00 | £301.00 | £43.00 |  |
| GP-Nurse | £19.00 | £0.00 | £0.00 | £95.00 |
| Psychologist/Counsellor consultation | £48.50 | £145.50 | £0.00 | £1,309.50 |
| **Cost to NHS** |  | £446.50 | £43.00 | £1,404.50 |
| IV.2. Travel & Other Costs |  |  |  |  |
| Travel cost for clinic visits |  | £18.75 | £28.15 | £504.00 |
| Other Expenses related to quit |  | £28.00 | £140.28 | £2,624.52 |
| Amount Reimbursed by the Study (NRT) | £29.30/person | £0.00 | £0.00 | £2,401.82 |
| **Net OOP expenses** |  | £46.75 | £168.44 | £726.70 |
| IV.3. Diagnostic tests |  |  |  |  |
| X-ray | £15.5 | £15.50 | £0.00 | £0.00 |
| Blood test | £6.5 | £0.00 | £0.00 | £6.50 |
| **Cost to NHS** |  | £15.50 | £0.00 | £6.50 |
| IV.4. Medicine Out-of-Pocket Expenses |  |  |  |  |
| Prescriptions payment | £9.0 | £27.00 | £0.00 | £2,322.00 |
| OOP expenses on non-prescription items |  | £2,165.00 | £1,413.00 | £559.00 |
| IV.5. Other Costs |  |  |  |  |
| Sick day cost due to stop smoking attempt | £99.2 per day |  |  | £99.20 |
| **V. Total Resource Use Cost** |  | **£2,700.75** | **£1,624.44** | **£7,519.72** |
| **VI. Total Cost of ntervention + Supplies + Resource Use** |  | **£16,174.35** | **£14,565.84** | **£20,247.72** |
| VI.1. Cost borne by Research Study |  | £13,464.60 | £12,925.90 | £2,401.82 |
| VI.2. Cost to Stop Smoking Services |  | £471.00 | £43.00 | £14,004.00 |
| VI.3 Cost to Participants (OOP expenses excluding reimbursement) |  | £2,238.75 | £1,596.94 | £3,841.90 |
| **VII. Per Participant Cost: Total Cost** |  | **£192.55** | **£177.63** | **£246.92** |
| Study Cost |  | £160.29 | £163.62 | £29.65 |
| NHS/Stop Smoking Services Cost |  | £5.61 | £0.54 | £172.89 |
| Participants' Cost |  | £26.65 | £20.21 | £47.43 |
| **Aggregated Total Cost per Participant** | **£208.97** | | | |

| **Incremental Cost-Effectiveness Ratios Nicotine-containing e-cigarettes vs Nicotine-free-cigarettes vs NRT** | | | |
| --- | --- | --- | --- |
| **Cost/QALY per Participant** | **Nicotine-containing e-cigarettes (EC-NR)** | **Nicotine-free-cigarettes (EC-NF)** | **Nicotine replacement therapy (NRT)** |
| 1. Cost of Stop-smoking Intervention borne by study | **£160.29** | **£163.62** | **£29.65** |
| 2. Stop Smoking Intervention + NHS Resource Use Cost | £5.61 | £0.54 | £172.89 |
| 3. Participants' Cost | £26.65 | £20.21 | £47.43 |
| **All Costs per Participant** | **£192.55** | **£184.38** | **£249.97** |
| QALY- Baseline | 0.8910 | 0.8840 | 0.9282 |
| QALY- 3M | 0.9118 | 0.9048 | 0.9531 |
| QALY- 6M | 0.9141 | 0.9103 | 0.9526 |
| QALY Difference 3M over Base | 0.0208 | 0.0208 | 0.0249 |
| QALY Difference 6M over Base | 0.0231 | 0.0263 | 0.0244 |
| **Incremental QALY Gain** | EC-NR over EC-NF | 0.0124 |  |
|  | EC-NR over NRT | | -0.0915 |
|  |  | EC-NF over NRT | -0.0791 |
| **Difference in Costs** | EC-NR over EC-NF | £8.17 |  |
|  | EC-NR over NRT | | (£57.42) |
|  |  | EC-NF over NRT | (£65.59) |
| **Incremental Cost-Effectiveness Ratio** | EC-NR over EC-NF | £659.86 |  |
|  | EC-NR over NRT | | £627.55 |
|  |  | E-NF over NRT | £829.13 |
